# The Potential Roles of ^**18**^F-FDG-PET in Management of Acute Stroke Patients

**DOI:** 10.1155/2013/634598

**Published:** 2013-05-15

**Authors:** Adomas Bunevicius, Hong Yuan, Weili Lin

**Affiliations:** Biomedical Research Imaging Center, University of North Carolina at Chapel Hill, CB 7513, Chapel Hill, NC 27599, USA

## Abstract

Extensive efforts have recently been devoted to developing noninvasive imaging tools capable of delineating brain tissue viability (penumbra) during acute ischemic stroke. These efforts could have profound clinical implications for identifying patients who may benefit from tPA beyond the currently approved therapeutic time window and/or patients undergoing neuroendovascular treatments. To date, the DWI/PWI MRI and perfusion CT have received the most attention for identifying ischemic penumbra. However, their routine use in clinical settings remains limited. Preclinical and clinical PET studies with [^18^F]-fluoro-2-deoxy-D-glucose (^18^F-FDG) have consistently revealed a decreased ^18^F-FDG uptake in regions of presumed ischemic core. More importantly, an elevated ^18^F-FDG uptake in the peri-ischemic regions has been reported, potentially reflecting viable tissues. To this end, this paper provides a comprehensive review of the literature on the utilization of ^14^C-2-DG and ^18^F-FDG-PET in experimental as well as human stroke studies. Possible cellular mechanisms and physiological underpinnings attributed to the reported temporal and spatial uptake patterns of ^18^F-FDG are addressed. Given the wide availability of ^18^F-FDG in routine clinical settings, ^18^F-FDG PET may serve as an alternative, non-invasive tool to MRI and CT for the management of acute stroke patients.

## 1. Introduction

In the Western society, stroke is the fourth leading cause of death and a major cause of permanent disability [1, 2]. Ischemic stroke is the most common type of stroke, comprising approximately 87% of all strokes [1]. The main goal of current acute stroke management is to prevent at-risk tissue from infarction by restoring blood flow to ischemic penumbral areas. Intravenous (IV) thrombolysis with recombinant tissue plasminogen activator (tPA) has been well documented offering improved outcomes in ischemic stroke patients who received tPA within 4.5 hours after symptom onset [3–7]. However, this narrow therapeutic time window has substantially limited the amount of all stroke patients receiving tPA. As a result, extensive efforts have been devoted to identifying a subgroup of stroke patients who may benefit from tPA beyond the currently approved therapeutic window and/or to developing more effective therapeutic interventions. Specifically, noninvasive neuroimaging methods have been widely implicated offering insights into the presence or absence of salvageable tissues (penumbra), which could be used, potentially, to extend the tPA therapeutic window beyond 4.5 hours. In contrast, the advent of intra-arterial thrombolysis and the introduction of endovascular clot retrieval devices (e.g., MERCI, Penumbra, and Solitaire) have shown great potential in improving the efficacy of vascular recanalization, which in turn may further extend the therapeutic window [8, 9]. While promising results in extending the tPA time window and utilizing new endovascular clot retrieval devices have been reported, both methods carry additional risks of mortality, morbidity, and serious complications, underscoring the critical role of careful patient selection [10].

In a clinical setting, the accurate determination of stroke onset (time since last seen normal), competent neurological examination, and noncontrast computed tomography (CT) scans to rule out intracerebral hemorrhage are vital for selecting ischemic stroke patients who may benefit from reperfusion therapies [3]. Despite significant advancements in neuroimaging, only non-contrast CT has a proven value for the management of acute stroke patients [3, 11]. Nevertheless, the search for imaging tools capable of providing insights into tissue viability continues to gain interest, which in turn may improve patient selection for reperfusion treatments beyond the currently approved therapeutic window for tPA. The presence of neuroimaging-based evidence of salvageable brain tissue beyond the currently recommended rt-PA therapeutic window further emphasizes the need for identifying such tools [12–15]. Consequently, it was suggested that the currently employed “time-to-treat” approach may not be ideal and should be replaced by “tissue-to-treat” [16]. Clearly, one of the prerequisites for the utilization of the “tissue-to-treat” approach is the availability of accurate neuroimaging-based surrogate markers capable of discerning tissue viability. Such imaging-based biomarkers may distinguish stroke patients who could potentially benefit from tPA beyond 4.5 hour, and/or from neuroendovascular treatments. The optimal imaging modality should be rapid, accurate, and readily available in a busy clinical setting and accompanied by automatic data analysis techniques [16]. 

**Figure 1:**
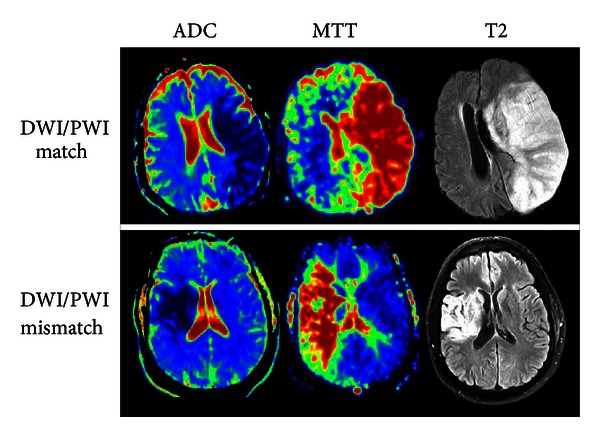
ADC and MTT maps demonstrating DWI/PWI match and mismatch in relation to the MRI T2 lesion.

In recent years, diffusion-weighted imaging/perfusion-weighted imaging (DWI/PWI) magnetic resonance imaging (MRI) [12, 15] and perfusion CT [17] have received considerable attention and have been suggested as imaging signatures for identifying the presence or absence of salvageable tissues. Specifically, the notion of diffusion/perfusion mismatch (DPM) has gained substantial interest as an effective tool to reveal the presence of ischemic penumbra [11, 12, 15, 18]. The underlying concept of DPM is that the abnormal *diffusion* areas represent the ischemic core, which will progress to infarction independent of treatments. In contrast, regions with abnormal *perfusion* represent tissues at risk of infarction if blood flow is not restored in a timely manner. As a result, brain regions with abnormal perfusion that reside outside of the abnormal diffusion areas, known as DPM, may be indicative of viable tissues, but they are at an increased risk of irreversible injury in the absence of timely reperfusion [19, 20]. Conversely, the absence of DPM would suggest no salvageable tissues.  Figure 1 shows acute diffusion/perfusion images as well as the final lesions from two representative patients. The upper row shows a patient with matched diffusion/perfusion deficits (imaged at 4.30 hours after onset and no tPA), suggesting the absence of salvageable tissues. Indeed, the acute diffusion/perfusion deficits spatially match with the final lesion. Acute DPM is observed in the patient shown in the bottom row (imaged at 1.19 hours with tPA), suggesting the presence of ischemic penumbra. Consistent with the DPM hypothesis, the mismatched region was not recruited into final infarction, demonstrating the potential clinical utility of DPM in an acute ischemic stroke. However, despite these promising results [12, 15], unacceptable reliability due to acute reversal of DWI lesions and the failure of all lesions to evolve to the final infarct have substantially limited clinical acceptance of DPM [16, 21, 22]. In addition, there is a lack of consensus on the choices of DWI and PWI thresholds delineating diffusion and perfusion abnormalities [23]. Therefore, a well-controlled clinical trial is needed to rigorously determine the clinical values of DPM prior to its routine clinical applications [24]. Although perfusion CT has been employed to identify patients for reperfusion therapies, it is limited by the need for an IV contrast injection, an additional radiation dose, and has less reliable threshold values when compared to DWI/PWI MRI [23, 25, 26]. 

**Figure 2:**
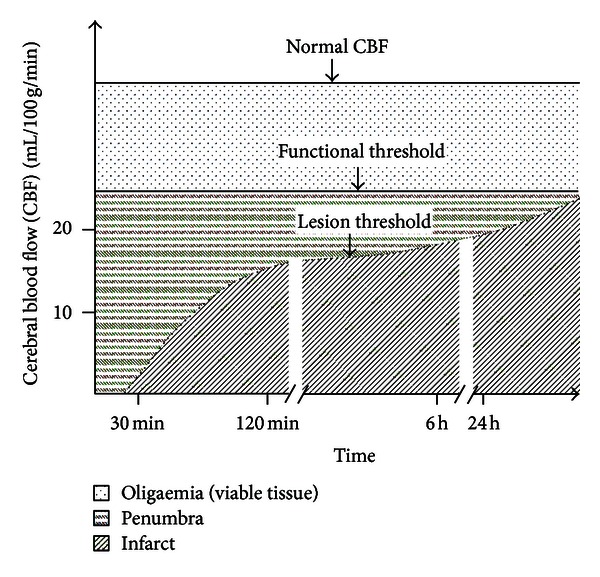
The association of cerebral blood flow thresholds and duration of ischemia with functional and structural tissue fates (adapted from [111, 112]).

In addition to MR and CT, PET has also been employed in an attempt to provide insights into brain tissue viability. This is perhaps not surprising, given its ability to provide *in vivo* measures of oxygen and glucose metabolism, the two main energy substrates of the brain. With ^15^O-labeled tracers, quantitative measurements of cerebral hemodynamics (cerebral blood flow (CBF) and cerebral blood volume (CBV)) and oxygen metabolism (oxygen extraction fraction (OEF) and cerebral metabolic rate of oxygen utilization (CMRO_2_)) can be obtained using PET. In particular, a number of seminal PET studies have demonstrated the existence of critical CBF thresholds and duration (Figure 2) below which functional and metabolic processes are disturbed and eventually cease [14, 18, 19, 27–30]. However, CBF alone cannot faithfully predict tissue fate; the duration to which neurons are under compromised perfusion is equally critical for final neuronal fate as shown in Figure 2 [27, 30, 31]. Furthermore, functional CBF threshold values have been shown to vary across neuron populations [30]. Regarding OEF, clinical and preclinical studies have demonstrated that areas with compromised CBF but increased OEF may be indicative of ischemic penumbra [32–35]. However, the final tissue fate of high OEF regions has been variable, suggesting that OEF is not a reliable marker of penumbral tissue [33, 35–37]. Finally, CMRO_2_ has been suggested as a highly promising parameter in predicting tissue fate in both experimental [38, 39] and human [32, 36] stroke studies. Specifically, it has been shown that brain regions with reduced CBF, but preserved CMRO_2_, are associated with neuronal survival, whereas brain regions with reduced CBF and CMRO_2_ likely reflect irreversible tissue damage [29, 32, 36, 38–40]. Collectively, while PET with ^15^O-labeled tracers provides essential physiological information that could predict tissue final fate during acute cerebral ischemia, the requirement of an in-house cyclotron to produce ultra-short half-life ^15^O-labeled tracers (~2 min) significantly hampers its clinical utility.

Alternatively, fluorine-18 (or ^18^F) has a half-life of 110 min, and [^18^F]-fluoro-2-deoxy-D-glucose (^18^F-FDG) has been utilized extensively to reveal *in vivo* glucose utilization in different clinical settings, most notably in oncology and cardiology. In myocardial infarction patients, PET with ^18^F-FDG has been routinely applied to discern viable myocardium (hypoperfused or stunned myocardium with preserved glucose metabolism), before considering coronary artery bypass graft surgery [41–43]. Along those lines, it is highly plausible that ^18^F-FDG may also offer valuable insights into cerebral glucose metabolism during cerebral ischemia, which consequently discerns tissue viability. In fact, there has been tremendous interest in evaluating the potential of ^18^F-FDG in cerebral ischemia. The recent innovation of the hybrid PET/MR scanners [44] may further renew interest on the potential clinical utility of PET with ^18^F-FDG in the management of acute stroke patients. To this end, this review paper will first provide an overview of glucose metabolism, followed by a comprehensive review of both animal and human studies utilizing ^18^F-FDG in ischemic stroke. Special attention is given to discussing temporal and spatial ^18^F-FDG uptake patterns during cerebral ischemia. Finally, the possible underlying biological mechanisms associated with the reported temporal and spatial ^18^F-FDG uptake patterns will be discussed.

## 2. Cerebral Glucose Metabolism

Maintaining normal brain homeostasis is an energy-consuming process that depends on a continuous supply of oxygen and glucose, since the brain cannot store energy substrates. Despite its relatively low weight (about 2% of the body weight), the brain receives 15% of the total cardiac output, 20% of the total oxygen consumption, and 25% of the total glucose utilization. The majority of energy in the brain (87%) is consumed for signaling; only about 13% of its total energy consumption is deferred for maintaining resting membrane potential [14]. 

**Figure 3:**
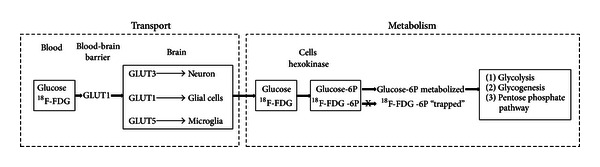
Transport and metabolism of glucose and ^18^F-FDG.

Normally, glucose is the primary metabolic substrate of brain cells. However, under certain conditions, brain cells can utilize other substrates, such as lactate, pyruvate, glutamate, and glutamine, which are endogenously synthesized and require glucose as their source of carbon. Under normal conditions, the glucose extraction fraction is approximately 10% [45, 46]. To enter brain cells, blood glucose is transported across the blood-brain barrier (BBB) and then across the plasma membranes of neurons and glial cells. This energy consuming process is mediated by glucose transporter (GLUT) proteins (Figure 3). Three major isoforms of GLUTs are considered important for glucose delivery to brain cells: GLUT1 is the primary glucose transporter in the blood-brain barrier, choroid plexus, ependyma, and glial cells and is not sensitive to insulin; GLUT3 is the predominant glucose transporter in neurons; GLUT5 is highly expressed in microglia [47]. Other types of GLUTs (2, 4, and 7) are expressed in lesser amounts and in more discrete brain regions [47]. When in cells, glucose is phosphorylated to glucose-6-phosphate (glucose-6P) by an enzyme, hexokinase. Once phosphorylated, glucose-6P is unable to exit the cell via the GLUTs and is trapped. Hence, phosphorylation of glucose is critical for the maintenance of the glucose concentration gradient across membranes ensuring a constant flow of glucose into cells via GLUTs [48]. Phosphorylation is also the rate-limiting step of glycolysis. Subsequently, glucose-6P can be processed into three metabolic pathways: (1) glycolysis; (2) glycogenesis; (3) pentose phosphate pathway. Glycolysis gives rise to two molecules of pyruvate, ATP and NADH. Pyruvate can then enter mitochondria and undergo the tricarboxylic acid (TCA) cycle and oxidative phosphorylation to produce 30 to 34 ATP molecules, CO_2_, and water. Oxygen is critical for the TCA cycle and oxidative phosphorylation. In healthy brains, OEF ranges from 30% to 40%, and CMRO_2_ averages from 3.0 to 3.8 ml/100 g tissue/min [27, 45, 46]. Finally, minute quantities of glucose-6P can be processed via the pentose phosphate pathway producing NADPH (a reducing equivalent), and glucose-6P can also undergo glycogenesis in astrocytes.

## 3. Evaluation of Glucose Metabolism in Ischemic Stroke

This section will provide a comprehensive overview of representative studies where glucose metabolism was employed for the study of ischemic strokes. A summary of these representative studies using ^18^F-FDG is provided in Table 1.

### 3.1. 2-Deoxy-D-Glucose (2-DG) Autoradiography in Ischemic Stroke

**Figure 4:**
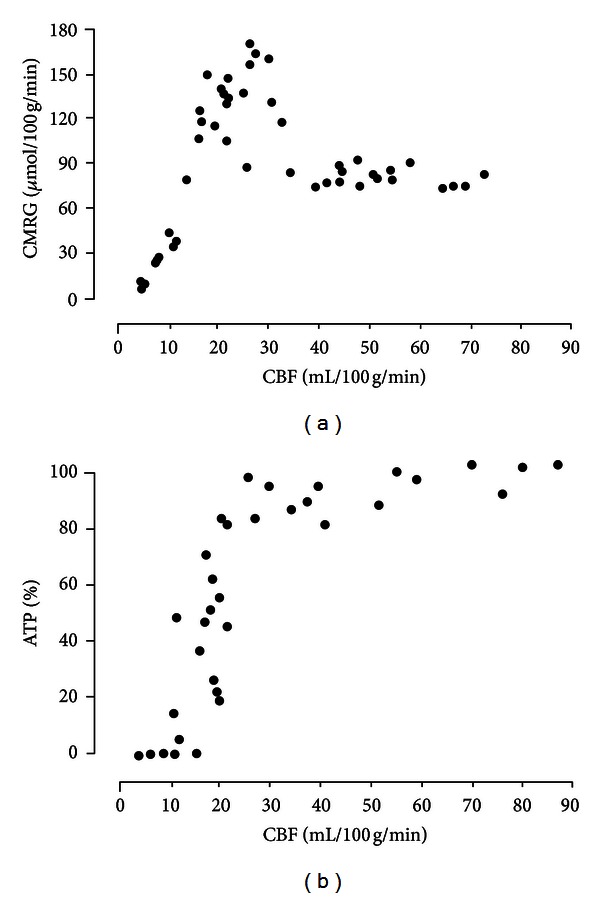
The association of cerebral blood flow (CBF) with cerebral metabolic rate of glucose (CMR_glc_) and ATP content in a gerbil stroke model (adapted from [50], with permission).

Early studies employed 2-DG as a surrogate marker of glucose metabolism. The 2-DG is a glucose analog with the 2-hydroxyl group replaced by hydrogen. Like glucose, the 2-DG is taken up by cells via GLUTs and phosphorylated by hexokinase. The 2-DG-6-phosphate cannot be further metabolized and is trapped in cells. The 2-DG is commonly marked with carbon-14 (^14^C) and is used in animal models to assess 2-DG distribution by means of autoradiography. Using ^14^C-2-DG, Ginsberg and colleagues demonstrated regions with decreased ^14^C-2-DG uptake in the presumed ischemic core. More importantly, their results showed a concurrent increase of ^14^C-2-DG uptake around the border of the ischemic core 60 minutes after a middle cerebral artery occlusion (MCAO) [49]. This unexpected finding could be of critical importance, particularly if the hyper ^14^C-2-DG uptake regions represent probable ischemic penumbrae. Paschen et al. further investigated the relationship between blood flow, glucose metabolism, and energy status in a permanent gerbil MCAO model [50]. They found that normal CMR_glc_ was maintained at rCBF > 40 ml/100 g/min but markedly increased at rCBF from 35 to 20 ml/100 g/min, sharply reduced, and eventually ceased at rCBF < 20 ml/100 g/min (Figure 4). Importantly, tissue ATP content was normal until rCBF fell below 20 ml/100 g/min. Comparing the relation between CMR_glc_ versus CBF and ATP versus CBF, it is evident that the compensatory increase of CMR_glc_ at rCBF between 20 and 35 ml/100 g/min was sufficient to replenish cellular energy content, leading to unsalted ATP even with compromised CBF. This elevation of glucose metabolism in regions with compromised CBF can potentially have profound clinical implications and serve as an imaging signature of ischemic penumbra. 

The utilization of ^14^C-2-DG for probing *in vivo* glucose metabolism is limited by its inapplicability for human studies, as well as for longitudinal evaluations of glucose metabolism. Thus, noninvasive markers of glucose metabolism are important for the assessment of temporal and spatial changes of glucose metabolism in ischemic stroke.

### 3.2. ^18^F-FDG PET in Ischemic Stroke

^18^F-FDG is a glucose analog in which the normal hydroxyl group in position 2′ is replaced by a positron-emitting radioactive isotope ^18^F [51]. Like glucose, the ^18^F-FDG is transported by GLUTs and is phosphorylated by hexokinase into ^18^F-FDG-6-phosphate (^18^F-FDG-6P) (Figure 3). The majority of ^18^F-FDG is trapped in brain cells ~1 hour after the injection, and thus PET scanning is usually initiated 40 minutes after the injection. The lack of the hydroxyl group prevents the ^18^F-FDG-6P from further metabolism and is thus trapped in cells. For this reason, the ^18^F-FDG-6P serves as a good marker revealing the *in vivo* distribution of glucose uptake by cells. Under normal physiologic conditions, neurons residing in the cortical gray matter, basal ganglia, cerebellum, and brain stem have the greatest glucose demand, resulting in the most intense ^18^F-FDG uptake [52, 53]. As mentioned previously, one of the major advantages of ^18^F-FDG is its long half-life, ~110 min. Therefore, an onsite cyclotron is not needed. With its long half-life, ^18^F-FDG has been widely used in experimental neurosciences and is the most commonly used radioisotope in clinical settings.

In the context of this review paper, we will focus our discussion on how ^18^F-FDG has been employed in the study of cerebral ischemia, particularly in predicting tissue fate. Experimental stroke studies will first be discussed, followed by patient studies. We will also determine if a similar elevated glucose metabolism in peri-ischemic regions as reported by Gingsberg et al. and Paschen et al. using ^14^C-2-DG was also reported using ^18^F-FDG.

#### 3.2.1. Animal Studies

^18^F-FDG has been widely used in experimental stroke research for more than two decades. However, early studies did not specifically investigate how ^18^F-FDG uptake and metabolism can be used to discern ischemic penumbra [32] and thus they will not be discussed here.

Two recent studies evaluated ^18^F-FDG metabolism 75 minutes [54] and 3 hours [55] after MCAO using an ischemic stroke model, respectively. Sobrado et al. carried out a longitudinal evaluation of ^18^F-FDG metabolism and infarct size [55]. Both permanent (right MCA + bilateral ICA occlusion followed by reperfusion of contralateral CCA after 75 mins) and transient (right MCA + bilateral ICA occlusion followed by reperfusion of all 3 vessels at 75 mins) ischemia were studied. MRI was acquired at 3, 24, and 48 hours after MCAO [55]. They found that relative to the contralateral hemisphere, areas corresponding to ischemic core had decreased ^18^F-FDG uptake in both transient and permanent MCAO models for all three time points. To further determine if ^18^F-FDG could predict final tissue fates, abnormal ADC regions in the transient MCAO group were divided into two subcategories: progressed (recruited tissue) versus did not progressed (recoverable tissue) to final infarction at 24 hours. Although a reduced ^18^F-FDG uptake was observed in both categories, ^18^F-FDG uptake was significantly greater in recoverable tissue when compared to the recruited tissue, suggesting that ^18^F-FDG may provide a means of predicting final tissue fate. In addition to measuring ^18^F-FDG uptake, Walberer et al. further evaluated how quantitative measures of the rate constants of ^18^F-FDG uptake correlated with CBF and tissue fates in a rat embolic MCAO model [54]. Specifically, ^18^F-FDG K1 (^18^F-FDG transports from blood to the brain) and Ki (^18^F-FDG net influx rate constraint) kinetic constants were measured 75 min after stroke, and their ability in predicting final tissue outcome (MR T2 images) at 24 hours was evaluated. Two major findings were reported in this study. First, a strong correlation (*r* = 0.89) between K1 and rCBF 60 minutes after MCAO was observed, suggesting that K1 of ^18^F-FDG can be a reliable estimate of rCBF during the hyperacute phase of a stroke. Second, elevated Ki (preserved glucose consumption) and reduced K1 (reduced rCBF) were observed 75 min after stroke, suggesting the presence of viable tissues through a compensatory increase of glucose uptake and phosphorylation in the hypo-perfused tissue.

In contrast to utilizing ^18^F-FDG during hyperacute stroke, Kuge et al. investigated ^18^F-FDG uptake in a primate thromboembolic stroke model (autologous blood injection into the left ICA) 24 hours after MCAO [56]. They found that regions with a decrease of CBF and significant reduction of CMR_glc_ at 24 hours after MCAO were consistent with negative 2,3,5-triphenyltetrazolium chloride (TTC) staining indicating infarction [57]. More importantly, several areas surrounding the ischemic core had moderately decreased CBF (40%–80% of contralateral values) and increased CMR_glc_ at 24 hours, which corresponded to TTC positive staining.

More recently, Fukumoto et al. carried out a serial PET study (before and 1, 3, 7, and 14 days after stroke) using multiple radiotracers, including ^18^F-FDG for glucose metabolism, ^11^C-(R)PK11195 (peripheral benzodiazepine receptors) for neuroinflammation, and ^11^C-FMZ (central benzodiazepine receptor) for neuronal integrity in a photochemically induced thrombosis (PIT) MCAO rat model [58]. In the core, there was a significant reduction of ^18^F-FDG uptake in all study time points that was accompanied by an increased ^11^C-(R)PK11195 binding (suggesting neuroinflammation) at days 7 and 14 and reduced ^11^C-FMZ binding (suggesting neuronal loss) at days 7 and 14. On the other hand, ^18^F-FDG uptake in the peri-ischemic areas was comparable to the normal brain regions at days 1 and 3 and was significantly increased on days 7 and 14. The latter increase of ^18^F-FDG coincided with increased ^11^C-(R)PK11195 uptake at days 3 through 14. These findings suggest that the delayed increased FDG uptake in the peri-ischemic regions was largely attributed to inflammation. Furthermore, at poststroke day 7, there was a significant overlap between increased ^18^F-FDG and ^11^C-(R)PK11195 uptake on autoradiography, as well as increased Iba1 immunohistochemistry staining. Together, these findings imply microglial activation at post-MCAO day 7. Similar results were also reported by Rojas et al. [59] although neuroinflammation was more pronounced in the ischemic core when compared to penumbra at day 4 after stroke. The difference in animal models may account for the discrepancies between these two studies. Specifically, in the transient MCAO model, collateral circulation is significantly reduced by transient occlusion of both CCAs, possibly preventing activation of neuroinflammatory cascade in penumbral areas. Furthermore, reperfusion injury in the PIT model can damage microglial cells, leading to silenced neuroinflammation [58].

#### 3.2.2. Clinical Studies

Despite promising findings from experimental stroke studies suggesting the potential clinical utility of ^18^F-FDG in discerning viable brain tissues, the applications of ^18^F-FDG in acute stroke patients are surprisingly scant; the majority of ^18^F-FDG studies in human ischemic stroke have been conducted in subacute or chronic phases of stroke. Nevertheless, some interesting findings and indirect evidence of elevated ^18^F-FDG uptake in the peri-ischemic areas similar to observations in animal studies have been reported [40, 60]. Several representative studies are reviewed below.

In 16 hemispheric ischemic stroke patients, Heiss and colleagues carried out multitracer PET, including ^18^F-FDG, H_2_^15^O, ^15^O_2_, and C^15^O, at 6–48 hours (tp1, mean = 23 hours) and again 13–25 days (tp2, mean = 15.6 days) after stroke [40]. Probable core was defined as brain areas with the greatest reduction of CMRO_2_ and CBF, whereas border zones of peri-infarct tissues were defined as 2 rims of 7.65 mm (or 3 pixels) surrounding the infarct core. Each rim was divided into 4 sectors, rendering 8 peri-infarct regions per patient. Not surprisingly, a significant reduction of OEF, CMRO_2_, CBF, and CMR_glc_ in the infarct core relative to the contralateral mirror region was observed at tp1, followed by hyperemia at tp2. For the border zone areas, although a significant reduction of CMRO_2_, CMR_glc_, and CBF at tp1 was observed which continued to decrease at tp2, the authors noted that the observed changes were highly heterogeneous. Qualitative examination of the peri-infarct regions revealed different outcomes with metabolic derangements. Specifically, peri-infarct regions with a stable or increased CMRO_2_, OEF, CMR_glc_, and glucose extraction fraction (GEF) at 24 hours after stroke were not infarcted, while low CMRO_2_, OEF, CMR_glc_, and OEF were consistent with CT evidence of infarction 4 days after stroke. More recently, Nasu et al. conducted ^18^F-FDG PET, MRI, and CT in 24 ischemic stroke patients 1 to 7 days after stroke onset [60]. Colocalized reduction of ^18^F-FDG uptake and an abnormal MR was noted in 20 patients. Of which, hyper uptake of ^18^F-FDG around the areas of decreased ^18^F-FDG was noted in 7 patients. Unfortunately, final tissue fates of the hyperuptake ^18^F-FDG regions were not evaluated in this study. Finally, chronic ^18^F-FDG studies (days and months after ischemic stroke) in stroke patients were also reported by several groups, and a reduction of ^18^F-FDG in the infarct areas is consistently reported [32, 45, 60, 61].

**Figure 5:**
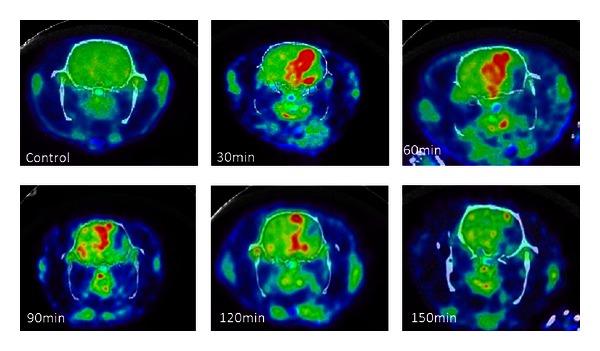
^18^F-FDG uptake prior to MCAO (control) and at different times after MCAO in a transient intraluminal MCAO rat model.

In summary, experimental studies of small animal [54, 55, 58] and primate [56] ischemic stroke models have revealed a consistent pattern of reduced ^18^F-FDG uptake in the presumed ischemic core regions. However, the temporal and spatial patterns of ^18^F-FDG uptake in the peri-ischemic regions are more variable in the literature. An acute elevated ^14^C-2-DG uptake in the peri-infarct area was reported by Ginsberg et al. [49]. Paschen et al. [50] further demonstrated that this increased uptake region corresponded to the ischemic penumbra. Interestingly, ^18^F-FDG studies conducted by Sobrado et al. [55] and Walberer et al. [54], both conducted during acute ischemia, failed to observe elevated ^18^F-FDG uptake in the peri-infarct area. Instead, an elevated uptake was observed at later times, >1 day after MCAO, which is more consistent with neuroinflammation. Attempting to discern the potential discrepancies in the temporal and spatial uptake patterns of glucose at the peri-ischemic region, we recently conducted ^18^F-FDG PET study using a transient intraluminal MCAO ischemic stroke rat model. ^18^F-FDG uptake patterns 30, 60, 90, 120, and 150 mins after MCAO were revealed. Final tissue fate was determined using 24 hours T2-weighted MR images. In concert to findings reported in the literature, we observed reduced ^18^F-FDG uptake in the ischemic core regions immediately after MCAO that remained low across all time points (Figure 5). Elevated ^18^F-FDG uptake in the peri-ischemic region was evident from 30 to 120 minutes, but largely diminished at 150 minutes after MCAO. More importantly, the majority of ^18^F-FDG hyper-uptake regions were not recruited in the final infarction at 24 hours, suggesting that the compensatory increase of ^18^F-FDG uptake may be associated with neuronal survival and is consistent with that reported by Paschen et al. [50]. Although additional studies are needed to further confirm our findings, our study suggests that acute elevated ^18^F-FDG uptake may offer a pathophysiologically relevant marker of tissue viability.

Similar to the experimental stroke studies, a severe reduction of ^18^F-FDG uptake at the presumed core area has also been consistently reported in human stroke studies [40, 45, 60, 61]. However, results on quantitative and qualitative temporal and spatial patterns of ^18^F-FDG uptake in stroke patients, particularly during the acute phase, are lacking. With ongoing technological advancements of PET imaging that allow more precise evaluation of spatial ^18^F-FDG uptake patterns, further studies specifically assessing ^18^F-FDG metabolisms in acute ischemic stroke patients are warranted.

## 4. Possible Mechanisms Underlying Increased ^18^F-FDG Uptake in the Probable Penumbral Tissue

In light of the potential clinical utility of the observed acute ^18^F-FDG hyper uptake in revealing the presence of ischemic penumbra, this section will discuss potential cellular and physiological mechanisms that may contribute to such ^18^F-FDG hyper-uptake at the peri-ischemic regions (Table 2).

### 4.1. Cellular Mechanisms

#### 4.1.1. Activation of GLUTs

As discussed previously, GLUTs play a critical role in transporting glucose from blood to the brain. Therefore, an upregulation of GLUTs expression may lead to an increased ^18^F-FDG uptake in PET images. A number of studies have reported that GLUTs can be up-regulated in response to cerebral ischemia in a manner similar to the observed temporal and spatial patterns of ^18^F-FDG uptake in ischemic stroke studies [54, 55]. Specifically, experimental studies of neuronal gene therapy have demonstrated that induction of brain GLUT1 overexpression during ischemic insult was associated with a significant increase of glucose transport using 2-DG autoradiography [62], as well as improved neuronal survival [63, 64]. Lee and Bondy reported that MCAO in rats induced a global and immediate (within an hour) increase of GLUT1 glial and neuronal mRNA expressions even in brain regions that normally do not express GLUT1 [65]. GLUT1 mRNA expression subsequently lateralized to the ischemic hemisphere and was mainly evident in the cortical regions surrounding the ischemic core at 24 hours. McCall et al. demonstrated an increased number of parenchymal and microvascular GLUT1s 24 hours and 4 days after MCAO [66]. Vannucci et al. studied changes of GLUT1 and GLUT3 mRNAs expressions after 2.5 hours of severe hypoxic-ischemic insult in 7-day-old rat brains [67]. They found elevated BBB GLUT1 mRNA expressions in both hemispheres 1 hour after ischemic insults. In addition, similar to observations by Lee and Bondy [65], this elevated GLU1 mRNA expression only persisted in the ipsilateral hemisphere during 24 hours of recovery. In the same study, the authors further reported that the temporal expression of neuronal GLUT3 mRNA appeared tissue fate dependent. The GLUT3 mRNAs expression continued to decrease in the core throughout the entire study, while the penumbral areas exhibited an increase of GLUT3 mRNA expression at 1 hour but started to decrease 3 hours after ischemic insults. Finally, maximally increased GLUT5 expression at the peri-infarct areas has been reported in a rat MCAO model 5 days after stroke that remained elevated until 15 days after the insult [68], suggesting that GLUT5 activation is consistent with neuroinflammation in response to neuronal necrosis [47].

In summary, temporospatial changes of GLUTs 1 and 3 mRNA expressions in ischemic conditions may partially account for acutely increased ^18^F-FDG PET uptake in the peri-ischemic regions [40, 54–56, 58, 60]. This up-regulation of GLUTs can serve as an important compensatory mechanism to facilitate glucose transport via the BBB and into cells in order to replenish energy stores and promote neuronal survival. In contrast, increased GLUT5 expression may be more associated with inflammation during the sub-acute phase.

#### 4.1.2. Activation of Hexokinase

Phosphorylation by hexokinase is one of the principal steps in ^18^F-FDG metabolism and is also a rate-limiting step of glucose metabolism. Up-regulation of phosphorylation in response to reduced oxygen availability has been suggested. Specifically, Paschen et al. demonstrated that a greater proportion of ^14^C-2-DG was phosphorylated in ischemic regions when compared to healthy brain regions (90% versus 80%) in an MCAO gerbil model [50]. As discussed previously, Walberer et al. demonstrated that part of the peri-infarct tissues had reduced K1 and increased Ki ^18^F-FDG rate constants 1 hour after MCAO, suggesting a compensatory increase of the glucose phosphorylation rate that requires both hexokinase and ATP [54]. Studies of brain tumor cell cultures and in myocardial infarction animal models reported upregulated hexokinase mRNA expression in response to hypoxia [69–71]. Similarly, a study of cerebrocortical cell cultures found that 3 days of exposure to 1% oxygen increased activities of glycolytic and its related enzymes (hexokinase, lactate dehydrogenase, and pyruvate kinase), as well as decreased activities of the TCA cycle related enzymes (citrate synthase and glutamate dehydrogenase), suggesting that neurons are capable of adapting to prolonged hypoxia by upregulating glycolysis and downregulating oxidative energy metabolism [72]. Finally, upregulation of hexokinase was observed in a global cerebral ischemic model using 7-day postnatal rats [73]. Collectively, these findings suggest that changes of hexokinase activity and expression in response to hypoxia may contribute to the increased “trapping” of ^18^F-FDG-6P in the peri-ischemic regions.

#### 4.1.3. Neuroinflammation

Neuroinflammation is a temporally and spatially dynamic process that includes resident brain cells (most importantly microglia) and blood-borne leukocytes and monocytes (for comprehensive review on neuroinflammation, see [74, 75]). It is generally believed that microglial activation is the first step of the neuroinflammatory process, followed by an influx of neutrophils 1 day after-stroke, and infiltration of macrophages 2 days after-stroke [74]. Since immune cells participating in acute (neutrophils) and chronic (macrophages) inflammatory responses have high metabolic demands, it may not be surprising that neuroinflammation results in an increased uptake of ^18^F-FDG [76–79].

Temporal and spatial progression of neuroinflammation was extensively studied in experimental stroke models. It was shown that mRNA expression of glial fibrillary acidic protein, a marker of reactive astrocytes, started to increase at 6 hours and continued to increase until day 3 after MCAO [80]. Using a permanent MCAO rat model, Mabuchi et al. found that activated microglia was evident in the peripheral area surrounding the infarction at 6 hours and continued to increase in number up to 48 hours after stroke, which coincided with a peak of macrophage accumulation along the boundary of infarction [81]. Furthermore, significant elevation of neuroinflammation 7 days after MCAO was demonstrated by an increased ^11^C-(R)PK11195 uptake in activated microglia cells as well as by increased number and activation of microglial cells and macrophages using immunohistochemical examination in ischemic penumbra [58, 82] and core [59].

In contrast to experimental stroke studies, temporal progression and spatial distribution of neuroinflammation in humans are most likely different [74]. Postmortem autopsy studies demonstrated the appearance of neutrophils at day 1 and peaked at days 2-3, as well as invasion of macrophages at day 5 (peak at 3 weeks) after stroke [83, 84]. However, post mortem studies are limited by their inability to provide temporal changes of neuroinflammation. In contrast, non-invasive imaging approaches have been employed to provide insights into the temporal behaviors of neuroinflammation [85–88]. Akopov et al. used SPECT to image 88 acute hemispheric stroke patients with technetium-99 m hexamethylpropyleneamine oxime-labeled leukocytes. An increased leukocyte accumulation was evident at 6 hours, progressively increased until 24 hours, and remained high up to 9 days after stroke [86]. Furthermore, ^11^C-PK11195 PET has been employed to study microglial activation [87, 88]. Price et al. performed ^11^C-PK11195 PET in 4 patients with left MCA territory strokes [88]. Significant binding potential of ^11^C-PK11195 was evident in the core and penumbral regions at 2 days and remained evident up to 30 days after stroke. The ^11^C-PK11195, however, lacks specificity to different subtypes of neuroinflammatory cells since it binds to mitochondrial peripheral benzodiazepine receptors that are expressed in astrocytes, macrophages, activated microglia, granulocytes, and lymphocytes [89, 90].

Activation of resident brain cells and transmigration of blood-borne immune cells can also be important determinants of the observed increased ^18^F-FDG uptake in the peri-infarct areas. However, to the best of our knowledge, there have been no studies systematically investigating temporal and spatial progression of *in vivo* neuroinflammation and its association with glucose metabolism in humans.

### 4.2. Physiological Associations

It is important to note that direct empirical evidence linking increased 18F-FDG uptake and to be discussed physiological mechanisms is currently lacking. Therefore, the proposed associations should be considered speculative rather than determinative. Nonetheless, we believe that it is important to discuss them because these mechanisms are physiologically relevant and are associated with cell activation that could lead to increased energy demand and thus ^18^F-FDG hyper-uptake.

#### 4.2.1. Peri-Infarct Spreading Depression-Like Depolarization

Peri-infarct spreading depression-like depolarizations (PIDs) are characterized as cortical DC shifts (about 20 mV) that spread along the cerebral cortex at a regular interval with a speed of 3–5 mm/min [91, 92]. Increased neuronal energy demands in PIDs could potentially be associated with ^18^F-FDG hyper-uptake. Results from experimental stroke models revealed that PIDs are triggered by the anoxic release of potassium and excitatory amino acids, tend to occur in clusters, and are associated with a significantly increased metabolic rate and energy demands that are not coupled with increase of blood flow. As a result, PIDs could lead to transient episodes of ischemia and the growth of an infarct core into the penumbral zone [91–93]. Therefore, it is plausible that PIDs induced neuronal activation, and thus the increased metabolic demand in the penumbral areas can be associated with the observed increased ^18^F-FDG uptake. For example, repeated cortical spreading depression (induced by cortical application of 3.3 M KCl solution) in cats was associated with increased cortical ^18^F-FDG uptake and rCBF in PET 60–120 minutes after the experimental procedure [57]. With regards to the temporal relation between PIDs and the onset of ischemia, studies in animal MCAO models indicated that PIDs occur immediately after stroke and progress until the terminal injury [94, 95].

Dohmen et al. were the first to demonstrate PIDs in ischemic stroke patients [96]. Specifically, electrodes were placed at the peri-infarct region of 16 patients undergoing decompressive craniectomy between 9 and 105 hours (mean = 39.8 ± 27.3 hours) after malignant MCA strokes. Spontaneous PIDs were recorded in all but two patients, who were later found to have electrode strips placed over the infarcted tissue. Interestingly, the frequency of PIDs (25 PIDs in 198 hours or 1 PID per 8 hours of monitoring) was the greatest in the patient with the shortest time interval between stroke onset and monitoring (13 hours). Not surprisingly, an increasing ECoG recovery time was observed over time, indicating progressive hemodynamic and metabolic deterioration. These findings suggest that PIDs seem to occur more frequently during acute strokes and decrease with time. PIDs were also recorded in humans with traumatic brain injury and intracerebral hemorrhage, albeit with a lower frequency than those in ischemic stroke patients [97], suggesting that these electrophysiological abnormalities are common in response to impeding functional and structural damage.

#### 4.2.2. Neuronal Regeneration

Neuronal regeneration starts early after ischemic insults and could also contribute to the observed increase of ^18^F-FDG utilization in the peri-infarct areas. For example, increased expression of mRNA encoding neuropilin-(Npn-) 1, Npn-2, and semaphorin 3A (Sema3A), proteins involved in axonal growth, was reported within hours after MCAO in rats [98]. Furthermore, mRNA expression of brain-derived neurotrophic factor, a member of the neurotrophin family promoting survival and growth of various nerve cell populations [99] and modulating glutamine excitotoxicity in penumbral areas [100], and its full-length receptor have been shown to increase in ischemic penumbrae and reduce in ischemic cores 12 hours after MCAO [101]. However, the temporal and spatial relationship between neuronal regeneration and glucose metabolism in ischemic stroke has not been fully explored to date.

## 5. Increased ^18^F-FDG Uptake versus Increased Glucose Metabolism

While the previous discussion offers some of the potential underlying biological mechanisms attributed to the observed hyper uptake of ^18^F-FDG in the peri-ischemic areas, one remaining major question is whether or not the increased ^18^F-FDG uptake truly reflects an increased glucose metabolism. To this end, it is important to discuss the lumped constant (LC), that is, the ratio of the metabolic rate of ^18^F-FDG and the metabolic rate of glucose. LC accounts for the differences in transportation, phosphorylation, and volume of distribution between ^18^F-FDG and glucose [102]. Therefore, an LC of 1 indicates that there are no changes between the metabolic rates of ^18^F-FDG and glucose; that is, the metabolic rate of ^18^F-FDG is equal to the metabolic rate of glucose. However, there are significant differences between phosphorylation of ^18^F-FDG and glucose. As a result, CMR_glc_ is usually estimated using a LC of 0.42. More recent studies in a cat MCAO model [103], and an *in vitro* cell model [104] separately demonstrated that ischemic or hypo-perfused tissues can result in a 20% to 78% increase of LC when compared to the normal brain tissue. These findings have profound implications on the utilization of ^18^F-FDG for assessing glucose metabolism. Specifically, in the event when concurrently increased ^18^F-FDG uptake and LC occurs, the increased ^18^F-FDG uptake might not reflect increased glucose metabolism. To make the matter worse, the extent to which LC is altered in response to ischemia can be time and species dependent [105]. Therefore, one must be cautious in the interpretation of ^18^F-FDG uptake as a marker of glucose metabolism.

## 6. Limitations of ^18^F-FDG PET

Despite its potential clinical values, limitations on using ^18^F-FDG PET for acute stroke patients should be acknowledged. Widely accepted standardized protocols for the acquisition and analysis of ^18^F-FDG PET remain lacking, limiting the quantitative evaluation of ^18^F-FDG PET across centers [106, 107]. Technical factors including optimal timing between ^18^F-FDG injection and PET imaging, the partial-volume effects [108], and uniform determination of ROIs should be considered [106]. Furthermore, the relatively long time interval between injection and PET imaging could make it impractical for the management of acute stroke patients. Radiolabeled tracers are sources of radiation, and the combination of PET with CT further increases radiation doses. Finally, ensuring 24/7 availability of ^18^F-FDG can impose challenges in some clinical centers.

Certain clinical situations that are common in the acute ischemic stroke settings can also dampen the clinical utility of ^18^F-FDG-PET. Transient hyperglycemia is common in acute ischemic stroke patients [109], which can impact the ^18^F-FDG PET results [107, 110]. Acute correction of hyperglycemia with insulin does not substantially improve ^18^F-FDG PET image quality because of different dynamics of normalization of plasma versus intracellular glucose concentrations [107]. Sedative medications that alter global metabolism of glucose should be considered. Finally, the inability of acute stroke patients holding still during PET images can also lead to compromised PET image quality.

## 7. Conclusions

Although ^18^F-FDG PET is widely available in clinical settings, its potential clinical utility for the management of acute stroke patients has not been extensively studied. Specifically, both experimental and clinical stroke studies have consistently demonstrated a reduction of ^18^F-FDG uptake in the presumed core regions, whereas the ^18^F-FDG uptake patterns in the peri-infarct regions are less consistent in the literature. This paper comprehensively reviews the temporal and spatial variations of glucose metabolism in response to cerebral ischemia with special attention on the peri-infarct regions. In particular, an elevated ^18^F-FDG (^14^C-2-DG) uptake in the presumed ischemic penumbra regions has been observed, which appears capable of predicting final tissue fate. The potential cellular mechanisms accounting for the increased glucose utilization and ^18^F-FDG uptake in the peri-infarct areas (activation of GLUTs, hexokinase, and neuroinflammation) were discussed. In addition, the possible physiological associations (PIDs and neuroregeneration) were proposed. Although the clinical utility of ^18^F-FDG PET in managing acute stroke patients remains to be seen, rigorous and systematic evaluations of ^18^F-FDG uptake patterns in acute ischemic stroke patients are warranted.

## Figures and Tables

**Table 1:** Published studies in experimental and human stroke studies evaluating ^18^F-FDG uptake.

Study	Stroke model/number of patients	Procedures	Poststroke timing	Relevant findings
Animals				

Walberer et al., 2012 [54]	Embolic MCAO in rats	^ 18^F-FDG PET MRI and histological examination (final infarct volume) ^15^O-H_2_O PET (CBF)	75 minutes 24 hours Before, and at 5, 30, and 60 minutes	(i) At 60 minutes, rCBF correlated positively with K1 (FDG transport from blood to brain). (ii) Infarcted tissue at 24 hours could be predicted by Ki (net influx rate constant) at 75 min. (iii) Parts of hypoperfused tissue that was infarcted at 24 hours had normal or elevated Ki at 1 hour.

Sobrado et al., 2011 [55]	Transient and permanent MCAO in rats	^ 18^F-FDG PET MRI (T2WI, DWI and PWI) Nissl staining	Before and at 3, 24, and 48 hours 3 hours	(i) ^18^F-FDG uptake in ischemic core regions was reduced for all time points after MCAO. (ii) At 3 hours after MCAO, areas that recovered with reperfusion at 24 hours had greater ^18^F-FDG uptake when compared to brain areas that progressed to infarction at 24 hours.

Kuge et al., 2000 [57]	Thromboembolic MCAO in primates	^ 18^F-FDG PET ^12^O-H_2_O PET (CBF) 2,3,5-triphenyltetrazolium chloride (TTC) staining	24 hours Before and 1, 2, 4, 6, and 24 hours 24 hours	(i) Ischemic core: reduced CBF, CMR_glc_, and negative TCC. (ii) Ischemic penumbra: moderate decrease of CBF, increase of CMR_glc_, and positive TCC staining.

Fukumoto et al., 2011 [58]	Thrombotic MCAO in rats	^ 18^F-FDG PET ^11^C-PK11195 PET (neuroinflammation) ^11^C-FMZ PET (neuronal integrity) ^11^C-PK11195; ^11^C-FMZ and ^18^F-FDG autoradiography Iba1 (microglia activation) and NeuN (neuronal damage) immunohistochemistry	Before and days 1, 3, 7, and 14 7 days	(i) Peri-infarct areas: significantly increased PET uptake of ^18^F-FDG at days 7 and 14 and of ^11^C-PK11195 at days 3, 7, and 14, plus Iba1 staining at day 7. (ii) Infarct core: reduced uptake of ^18^F-FDG at days 1–14, increased ^11^C-(R)PK11195 bindings at days 7 and 14 and reduced ^11^C-FMZ binding at days 7 and 14.

Humans				

Heiss et al., 1992 [40]	16 hemispheric stroke patients	^ 18^F-FDG, H_2_^15^O, ^15^O_2_, and C^15^O PET	6–48 hours 13–25 days	(i) Core: severely reduced OEF, CMRO_2_, CBF, and CMR_glc_. (ii) Penumbra: reduced CMRO_2_, CMR_glc_, and CBF. (iii) Some penumbral areas had increased CMRO_2_, OEF, CMR_glc_ and GEF and did not progress to final infarct. (iv) When compared to the first scan: Core—increased CBF; penumbra: reduced CMRO_2_, CMR_glc_, and OEF.

Nasu et al., 2002 [60]	24 ischemic stroke patients	^ 18^F-FDG PET, MRI, and CT	1–7 days Later time points	(i) In the acute phase ^18^F-FDG hyperaccumulation foci around hypoaccumulation areas were evident in 7 out of 20 patients. (ii) Final tissue fate of hyper-accumulation areas was variable.

**Table 2:** Possible mechanisms of increased ^18^F-FDG utilization in penumbral areas.

	*Cellular mechanisms*	Time course
Increased FDG transport		
GLUT1 upregulation	Increased ^18^F-FDG transport across the blood-brain barrier	Acute
GLUT3 upregulation	Increased ^18^F-FDG uptake by neurons	Acute
GLUT5 upregulation	Increased ^18^F-FDG uptake by microglia cells	Subacute to chronic
Increased FDG phosphorylation		
Hexokinase upregulation	Increased ^18^F-FDG-6P “trapping” in cells	Acute
Neuroinflammation		
Microglia activation	Increased ^18^F-FDG uptake by activated cells	Acute
Leukocyte migration	Increased ^18^F-FDG uptake by activated cells	Sub-acute
Macrophage migration	Increased ^18^F-FDG uptake by activated cells	Sub-acute

	*Physiologic associations*	

Peri-infarct speeding depression-like depolarization (PID)	Increased metabolic demand	Acute to sub-acute
Neuronal regeneration	Increased metabolic demand	Acute to sub-acute
